# Artificial intelligence in medical education: a cross-sectional needs assessment

**DOI:** 10.1186/s12909-022-03852-3

**Published:** 2022-11-09

**Authors:** M. Murat Civaner, Yeşim Uncu, Filiz Bulut, Esra Giounous Chalil, Abdülhamit Tatli

**Affiliations:** 1 Department of Medical Ethics, Bursa Uludag University School of Medicine, Bursa, Turkey; 2 Department of Family Medicine, Bursa Uludag University School of Medicine, Bursa, Turkey; 3 Institute of Health Sciences, Bursa Uludag University, Bursa, Turkey; 4 Bursa Uludag University School of Medicine, Bursa, Turkey

**Keywords:** Artificial intelligence, Healthcare, Medical curriculum, Medical ethics, Medical students, Medicine

## Abstract

**Background:**

As the information age wanes, enabling the prevalence of the artificial intelligence age; expectations, responsibilities, and job definitions need to be redefined for those who provide services in healthcare. This study examined the perceptions of future physicians on the possible influences of artificial intelligence on medicine, and to determine the needs that might be helpful for curriculum restructuring.

**Methods:**

A cross-sectional multi-centre study was conducted among medical students country-wide, where 3018 medical students participated. The instrument of the study was an online survey that was designed and distributed via a web-based service.

**Results:**

Most of the medical students perceived artificial intelligence as an assistive technology that could facilitate physicians’ access to information (85.8%) and patients to healthcare (76.7%), and reduce errors (70.5%). However, half of the participants were worried about the possible reduction in the services of physicians, which could lead to unemployment (44.9%). Furthermore, it was agreed that using artificial intelligence in medicine could devalue the medical profession (58.6%), damage trust (45.5%), and negatively affect patient-physician relationships (42.7%). Moreover, nearly half of the participants affirmed that they could protect their professional confidentiality when using artificial intelligence applications (44.7%); whereas, 16.1% argued that artificial intelligence in medicine might cause violations of professional confidentiality. Of all the participants, only 6.0% stated that they were competent enough to inform patients about the features and risks of artificial intelligence. They further expressed that their educational gaps regarding their need for “knowledge and skills related to artificial intelligence applications” (96.2%), “applications for reducing medical errors” (95.8%), and “training to prevent and solve ethical problems that might arise as a result of using artificial intelligence applications” (93.8%).

**Conclusions:**

The participants expressed a need for an update on the medical curriculum, according to necessities in transforming healthcare driven by artificial intelligence. The update should revolve around equipping future physicians with the knowledge and skills to effectively use artificial intelligence applications and ensure that professional values and rights are protected.

## Background

“Artificial intelligence” (AI) is a broad term that refers to technology that enables robots and computers to mimic human intellect [1]. While the information age is giving way to the era of artificial intelligence, certain professions, notably medicine, will be disproportionately impacted by this environment. AI technology is advancing at a breakneck pace and is transforming the realm of medicine, most notably via a process sparked by the Covid-19 epidemic. AI technologies are developed to analyze a variety of health data, including clinical, behavioural, environmental, and drug information, and data from biomedical literature as well as patients [1]. Apart from several other advancements, diagnosis and therapy can now be performed more quickly and precisely, imaging methods are improving, doctors and patients may be assisted by guiding surgery, drug research is facilitated, and more personalized therapies are feasible [2, 3]. Modern medicine generally takes a futuristic view of these identified difficulties. This futuristic tendency increases the appeal of AI applications in medicine, which look to be becoming more integrated into healthcare. The futurist author Topol’s words “Nearly every clinician in the future; from specialist physicians to paramedics, will be using artificial intelligence technology and especially deep learning.” underlined the wide field of use of AI in medicine [2]. On the other hand, there are numerous ethical concerns, including the threat to data security, the changing nature of the patient-physician relationship in health, the generation of potential social inequalities, and the development of AI robots that may eventually replace many professional tasks, resulting in increasing unemployment rates.

Healthcare providers are responsible for ensuring that AI applications provide useful technology in support of patient care. For this reason, gaining adequate knowledge and skills regarding AI applications in medicine is crucial for medical students, who may even have to use applications that did not exist during their education. Thus, the World Medical Association advocates for a review of medical curricula and educational opportunities for patients, physicians, medical students, health administrators, and other healthcare professionals to foster a better understanding of the numerous aspects of health care AI, both positive and negative [4]. Additionally, in a 2019 statement, the Standing Committee of European Doctors (CPME) stressed the need to use AI systems in basic and continuing medical education [5]. They proposed that AI systems be integrated into medical education, residency training, and continuing medical education courses to increase awareness of the proper use of AI. However, many authors in the literature stress that today’s medical education cannot meet the needs of AI and that a fundamental and compulsory change in education should be undertaken [2, 6–11]. Developing curriculum proposals specifically designed to train future physicians on AI would be a valuable contribution in that regard.

Understanding how today’s medical student perceives AI in medicine, what they know and don’t know, and their comprehension of AI’s ethical dimensions is a crucial first step to developing effective AI curricula. Kern et al. define one of the steps of curriculum development as “Needs Assessment of Targeted Learners”, a process by which curriculum developers identify the differences between the ideal and the actual characteristics of the targeted learner group [12]. Likewise, The CanMEDS Physician Competency Framework, a globally recognized framework that identifies the abilities physicians require to effectively serve, defines the needs assessment as identifying perceived and unperceived needs [13]. Grunhut et al. recommend that national surveys of medical students on the attitudes and expectations of learning AI in medical school should be carried out for developing curricula, and these surveys should identify the realistic goals physicians will be asked to meet, the expectations that will be put upon future physicians, and the resources and knowledge faculty members will need to meet these expectations [14]. Current studies in the literature fall short of a comprehensive needs assessment; they are limited in number and mainly focused on students’ knowledge and opinions on AI in medicine. The limited foci of the relevant studies can be categorized as follows;

- ‘Familiarity with AI’ (Pinto dos Santos et al. *(263 students / 3 medical schools in Germany)* [15], Bisdas et al. *(3133 students / 63 countries)* [16], Wood et al. (*121 students – 1 medical school in the USA)* [17], Oh et al. (*121 students - 1 medical school in Rebuplic of Korea)* [18], Blease et al. (*252 students – 4 medical schools in Ireland)* [19], Mehta et al. (*321 students – 4 medical schools in Canada)* [20], Sit et al. (*484 students – 19 medical schools in the UK)* [21]),
- ‘General thoughts of students on AI in medicine’ ([7, 16–18], Cho et al. (*100 students / 1 medical school in Rebuplic of Korea)* [22]),
- ‘Concerns about replacing physicians and losing jobs’ [15, 16, 18, 20–22],
- ‘Possible risks of AI in medicine’ [18],
- ‘Thoughts on the inclusion of AI in medical curriculum’ [15, 17, 19–22].

In summary, the findings conclude that future physicians are usually not familiar with AI, their concern of losing their jobs is considerable, they are enthusiastic to learn and use AI in their practice, and they think AI applications in medicine should be integrated into the curriculum. In addition, Bisdas et al. concluded that there might be a high demand to have AI topics integrated into the university curricula which should be further explored [16]. To the best of our knowledge, Wood et al.’s study with 117 medical students on seven topics is the only one in the literature that investigated the importance of AI topics in the eyes of the students. Hence, in this study, we aimed to examine medical students’ perceptions regarding the possible influence of AI on medicine and also their thoughts on the AI topics to be integrated into the medical curriculum.

## Methods

This multicenter study was conducted in a cross-sectional design using a web-based survey among Turkish-speaking medical students during the 2019–2020 academic year. The methodology was in accordance with the Checklist for Reporting Results of Internet E-survey (CHERRIES), as detailed below [23].

### Data collection

A survey form was constructed by the authors following a review of literature by searching Google Scholar (scholar.google.com), PubMed (pubmed.ncbi.nlm.nih.gov), and the Turkish Index Journal List (www.atifdizini.com) to define the items for the preliminary version. The survey consisted of three sections. The first section entailed demographic questions (school of medicine, age, gender, nationality, and year of medical education), past educational experience about AI, and participants’ self-evaluation of their knowledge of AI. The second section, including 15 five-point Likert questions focused on medical students’ perceptions of the possible influences of AI on medicine. Since no scale in the literature evaluates medical students’ perceptions of AI, a set of questions was prepared regarding the participants’ perceptions of the advantages and risks that AI would bring and the expectations of their future professional practices. In the beginning, there was a 28-items pool, which was limited to 15 items in the last version after considering three experts’ suggestions. In the third section, the participants were asked to state their thoughts on which topics about AI should be included in medical education. A pilot testing was applied to 30 medical students who did not take place in the developing process and the survey. Some items were revised according to the feedback. The online survey was designed and distributed by SurveyMonkey (www.surveymonkey.com). Ethical approval of the study was obtained from the Bursa Uludag University School of Medicine Research Ethics Committee (Date: May 27th, 2020 / Number: 2020–9/16). All participants were informed about the aim and nature of the study, their right not to participate or to quit the study when they would like to, and the data would be collected anonymously.

The reliability of the items on students’ perceptions on the possible influence of AI in medicine was calculated using Cronbach’s alpha, which was considered acceptable if the value was > 0.7. Besides, a split-half test was performed to detect any incongruence. The structure and subscales of the instrument were analyzed using explanatory factor analysis followed by direct rotation. As the extraction method, an Eigenvalue threshold of 1 was used. A minimum factor loading of 0.40 was taken as the criterion for each retained item. The Kaiser-Meyer-Olkin Measure of Sampling Adequacy and Bartlett’s Test of Sphericity were used to indicate the suitability of the data for structure detection. The internal reliability coefficients of the total items and dimensions of the scale were quite high. The Guttman split-half Coefficient was calculated as 0.754. The following table indicates the reliability of scales in terms of the value of Cronbach alpha as 0.7 which means that scales adopted for data collection are reliable and can be used for the study (Table 1).

**Table 1:** The internal reliability coefficients of the total items and dimensions of the scale

Variables	Items	Cronbach Alpha
Knowledge and Trust	5	0.793
Disadvantages and Risks	5	0.816
Informed Self Control	2	0.718
Total Items	12	0.841

The Kaiser-Meyer-Olkin Measure of Sampling Adequacy value for the obtained substance structure was calculated as 0.869. The Bartlett’s Test of Sphericity Chi-Square value was 8421.183, and the *p*-value was < 0.001. As a result, a factor structure consisting of 12 items and 3 dimensions, which could explain 60.6% of the total variance was obtained as shown in Table 2.

**Table 2:** Total variances explained by individual components

Items	Initial Eigenvalues		
	*Total*	*Percentage of Variance Explained*	*Cumulative Percentage*
1	4.477	37.306	37.306
2	1.804	15.035	52.341
3	1.002	8.354	60.695
4	.868	7.232	67.927
5	.672	5.598	73.525
6	.546	4.548	78.074
7	.533	4.444	82.518
8	.502	4.185	86.703
9	.473	3.944	90.647
10	.429	3.575	94.222
11	.386	3.217	97.440
12	.307	2.560	100.000

The Pattern Matrix of the factor structure of the Perceptions on the Artificial Intelligence in Medicine (PAIM) scale is shown in Table 3. It is noteworthy that the factor loadings of the items in the scale range between 0.401 and 0.844. Obtained factor dimensions were named as “Knowledge and Trust”, “Disadvantages and Risks,” and “Informed Self Control.”

**Table 3:** Pattern Matrix of the PAIM Scale’s factor structure

	Component		
	*Knowledge and Trust*	*Disadvantages and Risks*	*Informed Self Control*
1. Devalues the medical profession.		−0.793	
2. Reduces errors in medical practice.	0.800		
3. Facilitates patients’ access to the service.	0.704		
4. Facilitates physicians’ access to information.	0.760		
5. Enables the physician to make more accurate decisions.	0.715		
6. Increases patients’ confidence in medicine.	0.401		
7. Facilitates patient education.			0.844
8. Negatively affects the relationship of the physician with the patient.		−0.778	
9. Damages the trust which is the basis of the patient-physician relationship.		−0.811	
10. Reduces the humanistic aspect of the medical profession.		−0.771	
11. Violations of professional confidentiality may occur more.		−0.582	
12. Allows the patient to increase his control over his own health.			0.821

Since the response rate is generally low in web-based studies (ranging from 5 to 54%), it was aimed to reach all medical students all over the country [24]. There were 103 medical faculties and 95,035 students in the 2019–2020 academic year in Turkey (https://istatistik.yok.gov.tr). All medical students were invited to fill out the electronic survey via a message disseminated by the Turkish Medical Students International Committee (TurkMSIC) representatives in all medical schools. The survey was open for 3 months, with reminder messages sent at an interval of 1 month. Participation was voluntary and consent for study participation was obtained through the first page of the survey. Respondent anonymity was guaranteed by design. Multiple logins on the same IP address were blocked. A total of 3018 medical students from 67 medical faculties in all seven geographical regions of Turkey participated in the survey (The response rate was 3.17%). Participants who filled in the questionnaires incompletely were excluded from the study. Data for 2981 participants were analyzed.

### Statistical analysis

Responses on medical students’ perception on the possible influences of AI were collected using a Likert scale ranging from 0 (Strongly No) to 4 (Strongly Yes). Internal consistency was determined using Cronbach’s alpha and a split-half test. Test-retest reliability could not be conducted due to the anonymous data collection. Three items were removed after checking for internal consistency. Five of the final 12 items were reversely coded (items 1, 8, 9, 10, 11). Data were presented as n (%) or mean ± standard deviation (SD). Comparisons of the scores between the nationality groups were done with the independent samples t-test, while comparisons of more than two groups were made with the one-way ANOVA test. All analyses were conducted using the SPSS v20.0 software (SPSS Inc., Chicago, IL, USA). *p*-values < 0.05 were considered statistically significant.

## Results

### Opinions on the value of AI in medicine and perceived competence for utilizing AI

The mean age of the participants was 20.6 ± 2.4 years (min. 17, max. 40). The majority were women (59.5%), 38.7% were men, and 1.7% did not prefer to state their gender. A majority of the participants (87.9%) did not agree with the opinion that AI could replace the physician; instead, they thought that it could be an assistant or a tool that would help them. Nearly half of the students (44.9%) agreed that there would be a risk to lose their jobs with the decrease in the need for themselves. Three of every four participants (74.4%) agreed that they would become better physicians with the widespread use of AI. In addition, a quarter of the participants stated that their choice of specialization field would be influenced by how AI is used in that field (26.3%). Most of the students (75.6%) stated that they had not received any training on AI in medicine, while the other participants mentioned that they participated in some limited activities such as seminars and presentations, or received training over the internet.

Only 2.8% of the respondents answered that they felt knowledgeable about the use of AI in medicine, while one-third of the participants (35.2%) responded positively to the “Can you evaluate the reliability of a diagnostic application using AI?” question. Of all students, only 6.0% agreed that “I feel competent enough to inform patients about the features and risks of AI applications”. On the other hand, nearly half of the students stated that they could protect professional confidentiality when using AI applications (44.7%).

### Perceptions of the possible influences of AI on medicine

**Fig. 1:**
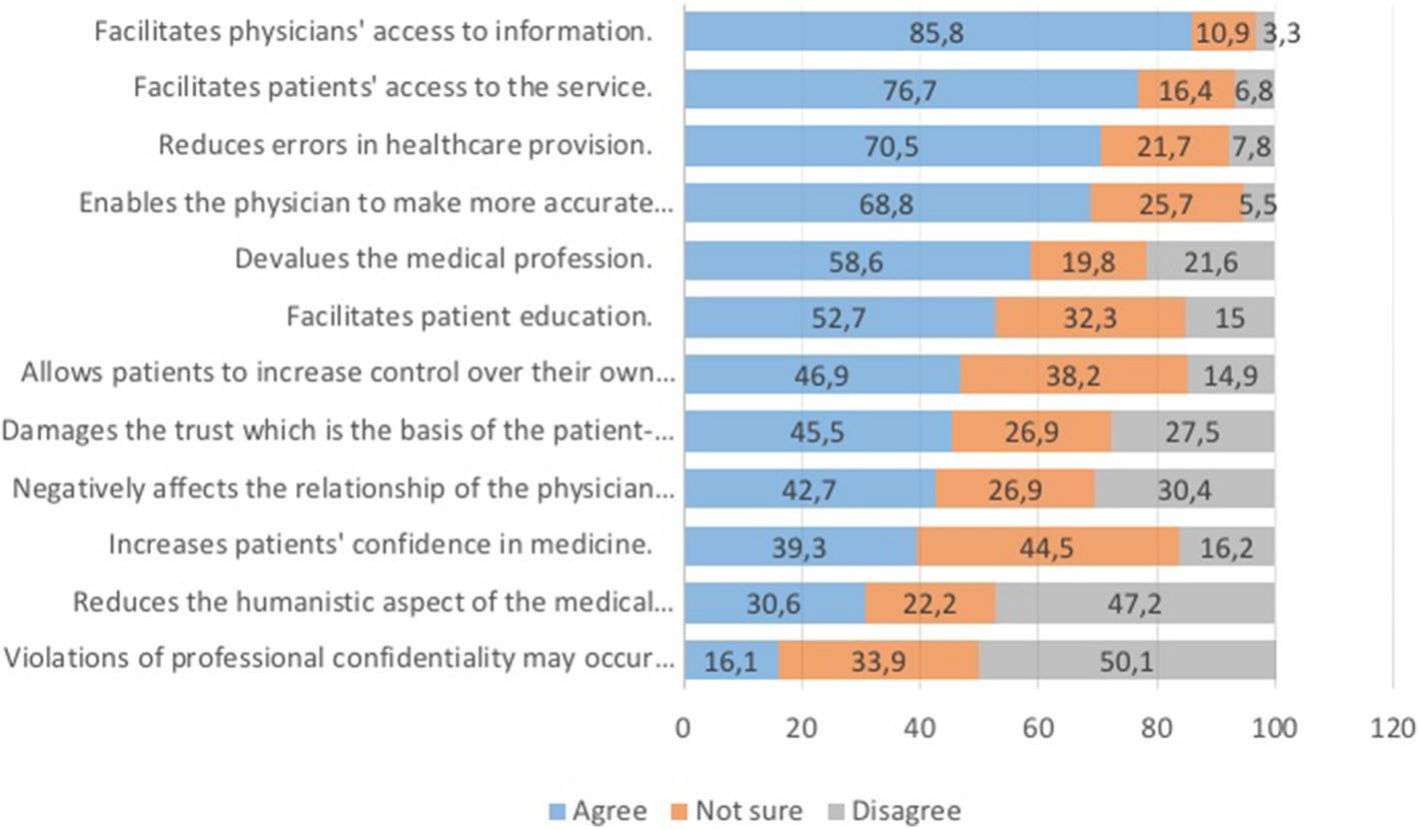
Descriptive analyses of the PAIM scale item responses (%)

Regarding student perceptions of the possible influences of AI in medicine, the highest agreement was observed on the item “Facilitates physicians’ access to information” (85.8%), while the lowest agreement was on “Violations of professional confidentiality may occur more” (16.1%) (Fig. 1).

The participants agreed more with the “Knowledge and Trust” subdomain than “Disadvantages and Risks” and “Informed Self Control.” The students were mostly in favour of AI in medicine because they felt it would facilitate physicians’ access to information (85.8%) and patients to healthcare (76.7%), reduce errors (70.5%), and help physicians make more informed decisions (68.8%). Participants were divided roughly in half in their considerations of the disadvantages and risks of AI in medicine: 58.6% agreed that AI would devalue the medical profession, 45.5% were concerned that using AI-assisted applications in medicine would damage the fundamental value of trust in patient-physician relationships, and 42.7% agreed that AI would negatively affect the relationship of the physician with the patient. Similarly, students were equally clustered on the positive and negative sides in the “Informed Self Control” subdomain: 52.7% agreed that AI would facilitate patient education, and 46.9% thought that it would allow patients to increase control over their health. There was no correlation between age and the PAIM scale scores (*p* > 0.05). Males had relatively higher mean PAIM scale and ‘Knowledge and Trust’ subscale scores. However, there were no significant differences between the scores of students in different years of study.

### On the need for an education

**Fig. 2:**
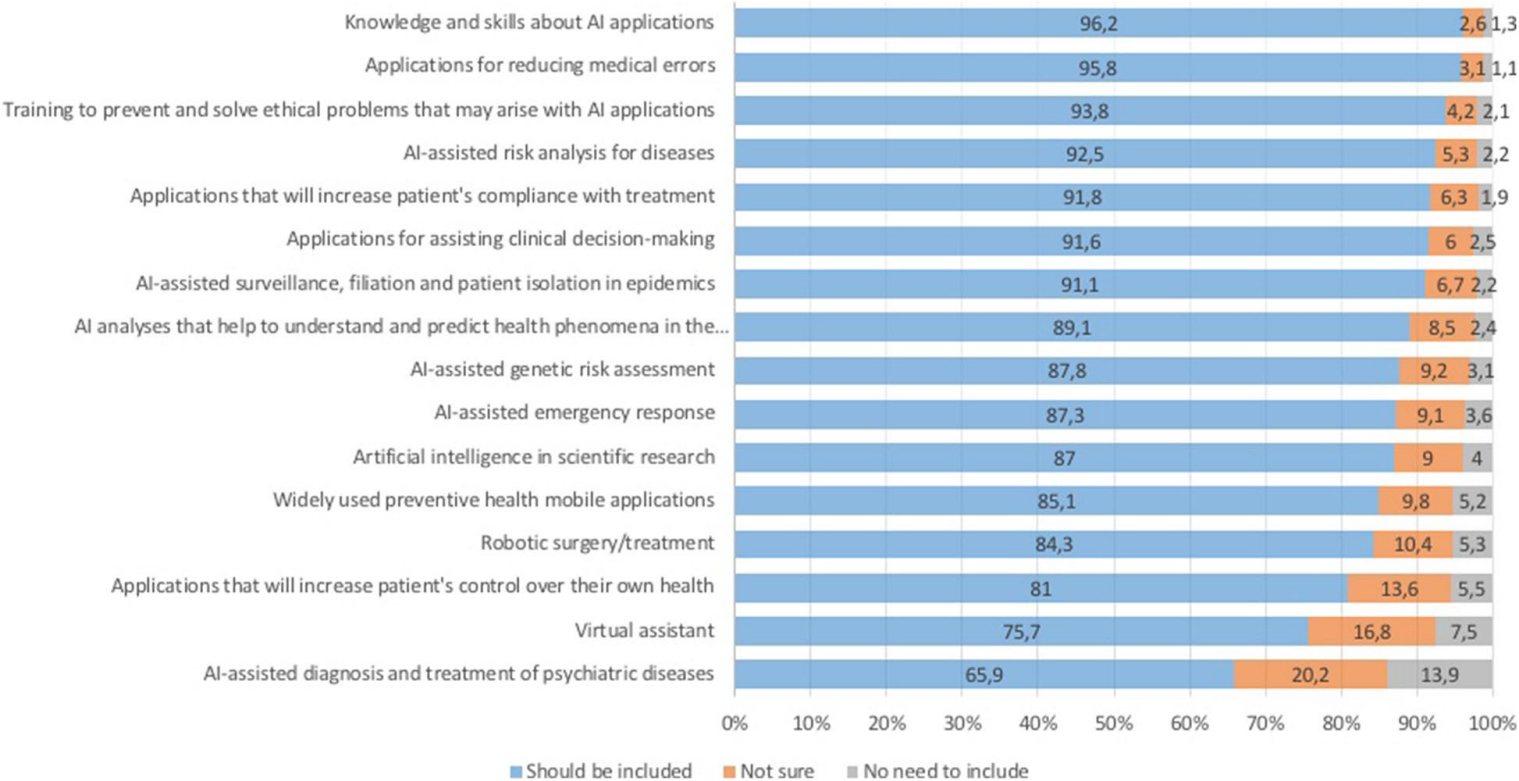
Opinions on the importance of topics that would be integrated into an AI education in medicine (%)

The vast majority of the participants were in favour of structured training on AI applications that should be given during medical education (93.8%). The participants think that it is important to be trained on various topics related to AI in medicine (Fig. 2). The most frequent topics that they perceived necessary in medical education were ‘Knowledge and skills related to AI applications’ (96.2%), ‘Applications for reducing medical errors’ (95.8%), ‘Training to prevent and solve ethical problems that may arise with AI applications’ (93.8%).

The participants also suggested various topics to be included additionally:

- A simplified lecture on Artificial intelligence, Computer use, Coding, Python language
- Criteria for selecting AI apps
- Mechanism of data leakage
- Conditions to be sought in the software in which the data will be recorded
- Assessing the reliability of AI apps
- Changing the value and necessity of the profession
- AI can only be used in addition to clinical decision/surgical skills and experience.
- International developments in AI
- How should communication with the patient be, what should be considered, and what are the possible risks regarding the usage of AI apps?
- Training on AI apps
- AI discussion sessions

## Discussion

### Opinions on the value of AI in medicine and its possible influences on medical practice

In this study, we examined the perceptions of future physicians about the possible influence of AI on medicine. In general, they were favourable and had high hopes for AI in medicine. They saw AI as an assistive tool that may improve doctors’ access to information, help physicians to make more accurate clinical judgments, minimize medical mistakes, and improve patients’ access to healthcare. Similarly, two-thirds of the students in a comparable study stated that AI developments would make medicine more exciting [16], and two-thirds in another study had positive attitudes toward the clinical use of the AI [18]. Regarding the PAIM scale scores, students’ perceptions were more positive in the ‘Knowledge and Trust’ subdomain than in the “Disadvantages and Risks” and “Informed Self Control” subdomains, which could be interpreted as being captivated by the excitement of a new promising technology while having substantial concerns at the same time. This is consistent with earlier research; concerns raised about AI in healthcare include confidentiality and privacy, patient safety, the impact on the profession’s humanistic components, and the rise of the commercialized medicine [2, 14, 17, 25–27]. The results varied according to gender, but not according to students’ year of study. We regarded the gender difference as indicative of men’s possible interest in technology. The absence of a difference between the study years validates the idea that contemporary medical education does not include any knowledge or understanding concerning AI.

A great majority of the respondents felt that AI could not replace physicians; instead, they thought that it could be an assistant or a tool to help them. Likewise, the majority of students surveyed in other studies perceived AI as a partner or a tool rather than a rival [16, 18]. Nevertheless, half of the students in this study were concerned about a reduced need for physicians and subsequent unemployment. This concern for the ways AI might negatively affect professional income and opportunity has been revealed in other studies as well, although their participants were less concerned, ranging between 29.3 to 38.6% [15–17, 28]. In addition to worries about personal opportunity and job security, it is not difficult to foresee that AI will have important effects on clinical care and therefore raises concerns about professionalism in medicine. Indeed, several scholars rightly argue that AI would be incapable of deep conversation and empathy toward the patient, which would cause distrust [18, 29]. Niet and Bleakley emphasized the intricate structure of clinical care based on clinical intuition and said that this could not be accomplished by technological care [30]. Mehta defined this amorphous quality of intuition as the “art of care” which he concluded was not possible with the AI [20]. The concern about a reduced need for physicians and unemployment might be a result of students’ unpreparedness for AI technology in medicine. Nearly half of the students believed that incorporating AI into medicine would devalue the profession, diminish its humanistic component, and erode confidence in patient-physician interactions. These are not negligible concerns. Instead of ignoring them as unjustified reactions, they must be addressed both by medical education and regulations aiming to protect those values and the fiduciary nature of the profession.

### On the need for education

We also examined the students’ education level on AI, and their thoughts on the need for specific educational topics to be integrated into the medical curriculum. The study revealed that the vast majority of participants did not receive a structured and consistent education about AI; only 2.8% reported feeling educated about the application of AI in medicine. Also, just one-third of respondents indicated a favourable response to the question “Can you assess the reliability of a diagnostic application using AI?”. Additionally, despite the future physicians’ responsibility to provide understandable and reliable information to their patients about AI applications in medicine [7, 14, 20, 30], just 6.0% of the participants felt qualified to inform patients about the features and hazards associated with AI technologies. Moreover, in terms of possible influences of AI in medicine, the lowest agreement was on the statement “Violations of professional confidentiality may occur more”. This is a remarkable finding, since protecting confidentiality is among the prominent concerns and one of the most important problem areas in the Big Data age. It is well known that healthcare data is one of the most valuable kinds of data since its abuse or breach could be very harmful to a person and society [31]. Failure to be aware of that kind of risk shows a serious need for a specific education for medical students. Participants’ overconfidence in protecting professional confidentiality and their feeling of incompetence regarding informing patients signify a need for education. Since Pinto dos Santos’s first research on what nation was published in 2019, similar results have been shown in investigations undertaken in other nations [15, 17–19, 21, 28]. In a recent review, Grunhut et al. wrote that “Students’ knowledge of AI is alarmingly low and insufficient to become future physicians” [14]. As Sapci and Sapci revealed in their systematic review, the integration of AI training into medical and health informatics curricula is an important need for future physicians [32]. A recently developed scale (MAIRS-MS) to measure medical students’ readiness for AI in medicine could provide a starting point in that regard [33].

### What to teach and how

Parallel to the lack of education and the feeling of incompetence, the students thought that AI should be part of the medical training, as was revealed in the other studies as well [15–17, 19]. Although there are some recommendations in the literature for curricular objectives [7, 8, 11, 14, 32, 34], Lee et al. concluded in their review that almost all curriculum recommendations lacked specific learning outcomes and were not based on a particular education theory [11]. Taking into consideration the medical students’ opinions could be useful for developing a consensus regarding desirable learning outcomes and appropriate educational theory. Wood et al.’s study with 117 medical students is the only one, to the best of our knowledge, that investigated the importance of AI topics in the eyes of the students [17]. In that study, medical genetics and genomics, radiology and digital imaging, individualized health data/device monitoring, and disease prediction models were regarded by the students as the most important ones among the seven topics. As a contribution to the findings of that study, we found that the students expressed a desire to gain specific knowledge and skills on many more topics related to AI, such as applications for assisting clinical decision-making and reducing medical errors, AI-assisted emergency response, and AI-assisted risk analysis for diseases.

In addition, we found that the students would also like to be trained on the ethical issuesnnthat may arise due to AI applications; they felt this was one of their most important topics. This expectation echoes Grunhut et al.’s question: “How can a physician untrained in the field of AI expect to navigate ethical scenarios such as if a computer algorithm predicts a high chance of death for a patient?” [14]. AI in medicine will inevitably raise new ethical challenges alongside the traditional ones, therefore the knowledge and skills to be able to prevent and solve ethical problems must be an essential part of any educational endeavour. In that regard, the AMEE guide on artificial intelligence in medical education recommends that “Complex issues already inherent in medical informatics’ ethics need to be built into medical AI as guiding principles. Only by including these ethical principles into AI, can AI move from Artificial Intelligence to Artificial Wisdom” [8]. Lee et al. summarized their review of the recommendations in the literature as “the ethical and legal implications of AI systems were considered essential in ensuring safe and informed use of AI systems, and specific learning objectives should include (1) frameworks to approach AI ethics and (2) facilitating discussions of important AI ethics topics like liability and data privacy”.

As for the methodology, Wartman and Combs defined an education model aimed at providing the ability to integrate and use information from increasing sources in an elective way, replacing the current medical education model, which is largely based on the rote learning [6]. According to Grunhut et al., the curriculum should be incorporated through previously proven methods in similar drastic curricular changes [14]. Cross-disciplinary courses, small-group sessions, experiential learning/providing opportunities for students to work directly with AI tools, e-modules, interactive case-based workshops, self-learning modules, and student site visits to learn about the creation of AI products are among the suggested methods to teach students AI basics and improve their understanding of AI ethics [11, 19, 34]. Multidisciplinarity is regarded as crucial while implementing those learning strategies. The AMA Council encourages the review of medical curricula and urges medical school deans to be proactive in recruiting non-clinicians such as data scientists and engineers [14]. Establishing partnerships with institutes across computer science, biomedical engineering, the basic sciences, and public health, organizing ‘hackathons’ and ‘datathons’ in collaboration with computer science and engineering students are suggested in the literature in that sense [11, 34].

Besides developing the content and the methodology of specific education, adapting to those changes could be one of the most important challenges for today’s medical educators. Grunhut et al. state medical school faculty simply have no understanding of how to implement these changes [14]. Therefore, educating educators seems a necessity to improve the traditional approaches and implement this growing set of recommendations.

### Limitations

The online survey method and voluntary participation require consideration of the possibility that the survey was completed only by students who were interested in the subject. However, the fact that students’ self-evaluations are compatible with other similar studies in the literature suggests that they put forward the problems in an impartial way. Although it is not possible to generalize the results to the country, we achieved a high number of participants from different regions. The 12-item questionnaire obtained at the end of the study gave the desired results in terms of factor analysis and validity. The absence of another measurement tool on this subject and the lack of re-testing in terms of the design of the study did not make it possible to conduct further validation studies. There is a need for studies designed in a more homogeneous sample.

## Conclusion

As AI technologies are increasingly being implemented in medicine, today’s medical students will be working in a different environment from the current one. This transformation has accelerated with the latest pandemic, and the need for a change in medical education and healthcare service delivery has emerged more profoundly. Yet students do not receive a structured or standardized education about AI (if they get any), which left them feeling ignorant and inadequate. With this study, we revealed students’ perceptions on the possible influences of AI on medicine and the profession, as well as their opinions on the topics that should be integrated into the medical curriculum. Therefore, our study contributes to needs assessment studies that are important for curriculum development and defining learning outcomes.

Medical students in Turkey are enthusiastic to learn how to use AI technologies while preventing ethical problems related to AI. This provides an invaluable opportunity for training physicians who respect the humanistic aspect of the patient-physician relationship and are able to protect professional values, especially in the age of Big Data, Artificial Intelligence, and commercialized medicine. We believe the right time to develop a common consensus on the core knowledge and skills AI demands, along with novel educational techniques to develop AI ethics and competency, is now.

## Data Availability

The datasets analyzed during the study are available from the corresponding author upon request.

## Abbreviations

AI: Artificial Intelligence
AMA: American Medical Association
AMEE: Association for Medical Education in Europe
MAIRS-MS: Medical artificial intelligence readiness scale for medical students
PAIM Scale: Perceptions on Artificial Intelligence in Medicine
CanMEDS: Canadian Medical Education Directions for Specialists
